# Effect of virtual versus traditional education on theoretical knowledge and reporting skills of dental students in radiographic interpretation of bony lesions of the jaw

**DOI:** 10.1186/s12909-019-1649-0

**Published:** 2019-06-25

**Authors:** Elham Soltanimehr, Ehsan Bahrampour, Mohammad Moslem Imani, Farshad Rahimi, Banafsheh Almasi, Marzieh Moattari

**Affiliations:** 1 Department of Pediatric Dentistry, School of Dentistry, Kermanshah University of Medical Sciences, Building No. 1, Kermanshah University of Medical Sciences (KUMS), Shahid Beheshti Boulevard, Kermanshah, Postal Code: 6715847141 Iran; 2 Department of Oral and Maxillofacial Radiology, School of Dentistry, Kermanshah University of Medical Sciences, Building No. 1, Kermanshah University of Medical Sciences (KUMS), Shahid Beheshti Boulevard, Kermanshah, Postal Code: 6715847141 Iran; 3 Department of Orthodontics, School of Dentistry, Kermanshah University of Medical Sciences, Building No. 1, Kermanshah University of Medical Sciences (KUMS), Shahid Beheshti Boulevard, Kermanshah, Postal Code: 6715847141 Iran; 4 School of Dentistry, Kermanshah University of Medical Sciences, Building No. 1, Kermanshah University of Medical Sciences (KUMS), Shahid Beheshti Boulevard, Kermanshah, Postal Code: 6715847141 Iran; 5 Nursing Department, Medical School, Islamic Azad University, Yazd Branch, Safaieh St, Yazd, Postal code: 8916871967 Iran

**Keywords:** Virtual education, Traditional education, Theoretical knowledge, Reporting skills, Radiographic interpretation, Bony lesions

## Abstract

**Background:**

This study aimed to compare the effect of virtual and traditional education on theoretical knowledge and reporting skills of dental students in radiographic interpretation of bony lesions of the jaw.

**Methods:**

This experimental study evaluated 39 dental students who had not received any instruction regarding radiographic interpretation of bony lesions of the jaw. They were randomly divided into two groups of virtual (*n* = 20) and traditional education (*n* = 19) and matched in terms of their grade point average (GPA). The virtual group received a virtual learning package while the traditional group received traditional classroom instruction for 6 one-hour sessions. Similar contents were taught to both groups by the same mentor. All students participated in a theoretical test with multiple-choice questions and an objective structured clinical examination (OSCE). Similar exams were also held 2 months later to assess knowledge retention. Data were analyzed using independent sample t-test and repeated measures ANOVA.

**Results:**

The mean scores of theoretical test (*P* < 0.05) and OSCE (*P* > 0.05) in the virtual education group (16.60 ± 0.91 and 15.13 ± 0.78) were higher than those in the traditional education group (14.89 ± 0.99 and 14.71 ± 0.92). In both methods of instruction, the mean scores of theoretical test and OSCE at 2 months were lower than the scores acquired immediately after instruction but this difference was not statistically significant (*P* > 0.05). Type of education had a significant effect on the theoretical test score (*P* < 0.001) but had no significant effect on the clinical score (*P* = 0.072).

**Conclusions:**

Virtual learning was superior to traditional lecture-based method for enhancement of knowledge acquisition in radiographic interpretation of bony lesions of the jaw. However, to improve our students’ reporting skills, we need to revise our virtual educational program so that the students have more opportunities to engage in reporting skills.

**Electronic supplementary material:**

The online version of this article (10.1186/s12909-019-1649-0) contains supplementary material, which is available to authorized users.

## Background

Effective education is believed to be the most fundamental factor in prosperity and knowledge of students. Learning must be independent and self-directed in order to be effective. Cooperation and interactions between students regarding the instructed topic are also important and the results of these interactions should last for long [1]. Evidence shows that students are more interested in challenging educational methods and would like to learn in an up-to-date and vibrant environment [1–3]. Thus, a balance should be maintained between the students’ demands and the educational system [2].

Mentors play a pivotal role in education, and their quality of instruction can significantly affect the students’ learning. Thus, there seems to be a need for an educational system, which is less affected by the individual problems and psychological status of the mentors. On the other hand, advances in medical and dental sciences in the recent decades have significantly increased the load of new topics to learn, and sufficient time is not available for this purpose in the traditional model of classroom education. As a result, the educational method has gradually changed to lecture-based instruction of theoretical topics, which is not interactive. Assessment of the quality of learning is also confined to written exams. Thus, the administrators and educational authorities have come to believe that the quality of learning of students is not satisfactory and are searching for strategies to improve it [3, 4]. Thiele et al. [5] evaluated nursing students and concluded that traditional classroom education is tiresome and the force to learn at a certain predetermined time limits actual learning. In contrast, learners have access to educational content at any time in virtual instruction, which increases the efficacy of instruction. Regarding the differences between the two methods of traditional and virtual instruction, a review by Potomkova et al. [6] discussed that medical students mainly preferred web tutorials compared with traditional lecture-based classes due to advantages such as easy accessibility, easy use, the freedom of navigation, the high quality of images and the possibility of repeating the practice.

Advances in science and technology have revolutionized the educational methods at different levels, and medical and dental education is no exception [7]. Considering the availability of computer and high-speed Internet, many major universities worldwide now offer long-distance learning, web-based learning, closed online learning and educational video programs and these methods are becoming increasingly popular in dental education as well [8]. The traditional medical and dental education was based on the attendance of mentor and students in a traditional classroom setting. However, this education can now be provided long-distance and can even be archived for later use. Evidence shows that students are more interested in using novel methods of education instead of traditional learning [9, 10]. Moreover, electronic learning can help the students to access the latest findings and employ evidence-based dentistry [11]. Accordingly, virtual education and online instruction are becoming increasingly popular worldwide. Virtual education is among the most important applications of information technology, which is offered in various forms of computer-based online and offline learning and network-based instruction [12]. The main advantage of virtual learning is that the learner can access the educational content anytime and anywhere with no limitation [12, 13]. However, considering the independent nature of this method, group discussion and communication with peers are not often feasible, which is a limitation of this technique. High cost of establishing a virtual instruction network and the need to have a computer and the necessary skills to use this system, inadequate computer knowledge and unfamiliarity with the copyright rules and regulations are among the limitations of this method of instruction [14].

Virtual and traditional education have differences in terms of efficacy and knowledge transmission [11, 15] and it is imperative to assess and compare the efficacy of these methods for instruction of different topics. Considering the nature of dental science and the need for visualization of educational contents, virtual instruction can be used at different levels for dental education. Virtual education of dental sciences may include educational films of long and complicated dental procedures, access to scientific resources such as textbooks, abstract books of congresses and articles, discussions related to clinical cases, photo-gallery, etc. [16]. Electronic learning programs have been used for instruction of various topics in dentistry, and many previous studies have evaluated the efficacy of virtual education for instruction of topics in oral and maxillofacial radiology [9, 17–20]. However, the efficacy of virtual education for teaching radiographic interpretation of bony lesions of the jaw has not been previously studied.

On the other hand, reporting skills comprise a large portion of educational curriculum for dental students. Instruction of reporting skills to dental students is mainly performed in preclinics and clinics and some concerns exist with regard to electronic instruction of these topics. Studies on the efficacy of virtual education for reporting skills are limited and this topic is in need of further investigation. Considering all the above, this study aimed to compare the effect of traditional and virtual education on theoretical knowledge and reporting skills of dental students in radiographic interpretation of bony lesions of the jaw to find the most effective method of instruction for this particular topic. The null hypothesis was that the effects of traditional and virtual education would not be significantly different on the theoretical knowledge and reporting skills of dental students in radiographic interpretation of bony lesions of the jaw.

## Methods

This experimental study was conducted in 2018 and evaluated 39 fourth-year dental students of Shiraz University, School of Dentistry who had not received any instruction regarding radiographic interpretation of bony lesions of the jaw. They had passed “radiology 1” and “radiology 2” courses. According to the educational curriculum of the Ministry of Health and Medical Education in Iran, Radiology 1 and 2 courses mostly focus on topics such as physics of radiation, biology of radiation, image acquisition and imaging errors. The Radiology 3 course is mainly about bony lesions of the jaw and their radiographic interpretation. All 4th year dental students were invited to participate in this study. Of 54 students that were invited, 39 willingly accepted to participate in the study and were enrolled. Minimum sample size was calculated to be 19 in each of the two groups according to a previous study by Moazami et al. [21] assuming 95% confidence interval, power of 80%, d = 4 and mean second post-test score of 3.35 and 4.88 in the traditional and virtual education groups, respectively.

Written informed consent was obtained from each participant. The study was approved in the ethics committee of Kermanshah University of Medical Sciences.

In this study, traditional teaching was defined as traditional lecture-based education in a classroom setting in presence of a mentor. This method is routinely practiced in all universities in Iran. Virtual teaching was defined as using a learning management system (LMS), which included a combination of facilities such as a learning path, quizzes, weekly homework, useful links, related articles and active interactions of students and mentors.

Participants were randomly divided into two groups of virtual (test group; *n* = 20) and traditional (control group, *n* = 19) education. The two groups were matched in terms of average mark received for all the courses a student takes over previous academic years which is known as grade point average (GPA), age and sex since Reime et al. [22] and Rosenfeld et al. [23] discussed that the efficacy of traditional and electronic learning is significantly associated with age and sex of students.

Classes were held for both groups during 6 weeks and the same educational content regarding radiographic interpretation of bony lesions of the jaw was taught to them according to the educational curriculum provided by the Ministry of Health and Medical Education. Prior to commencing the virtual education for the test group, an informatory session was held to instruct them on how to use the LMS. This group of students were allowed to use the LMS repeatedly during 6 weeks. During this time period, the test group students were provided with educational resources regarding radiographic interpretation of bony lesions of the jaw including multimedia contents with online and offline access such that students could access the contents whenever they wished to do so. The resources included PowerPoint presentations by the mentor that were in accordance with the sharable content object reference model, related textbooks and articles in PDF format and related links to other educational websites. Also, this system allowed students to communicate with each other or with their mentor. The LMS also had some homework for the students to do such that at the end of each session, radiographic images of bony lesions of the jaw (corresponding to the taught topic) were shown to students and they were requested to write a radiographic report for them during the week. The reporting phase was designed such that the student submitted his/her report, was able to read the reports of others only after submission of the report. Also he was not allowed to change his report after viewing others’. At the end of each week, the reports were corrected by the mentor and questions were answered. Students in the traditional learning group received 6 sessions of traditional classroom instruction, 1 h each, which were based on lecture and PowerPoint presentation. The same educational content was taught to students by the same mentor.

To assess immediate learning, all students participated in a theoretical test immediately after completion of instruction in both groups, which included 40 multiple-choice questions (Additional file 1). Also, their reporting skills were evaluated by an objective structured clinical examination (OSCE) and they were requested to write a radiographic report for the bony lesions (Additional file 2). All tests were evaluated by one faculty member. The theoretical test included multiple choice questions and the papers could be easily evaluated and scored. For the OSCE, a checklist was designed and the student reports were evaluated and scored according to the checklist. Also, the same exams (with the exact same questions) were held 2 months later to assess their knowledge retention.

The scores acquired by the students in each exam were analyzed. The validity of the tests was confirmed by the faculty members of the Oral and Maxillofacial Radiology Department of Shiraz University of Medical sciences, School of Dentistry. The reliability of the tests was assessed by KR20 test (https://methods.sagepub.com/Reference//encyc-of-research-design/n205.xml), which analyzed the results of previous exams held for another group of students (not participating in this study). R for the theoretical and clinical tests was found to be 0.73 and 0.79, respectively.

At the end of the study, students in the traditional group were also granted access to the virtual educational content. Moreover, students participated willingly in the study and were ensured about the confidentiality of their information.

### Statistical analysis

Data were analyzed using descriptive and inferential statistics. Mean and standard deviation were reported for descriptive data while normal distribution of inferential data was assessed using the Shapiro-Wilk test. Independent t-test and repeated measures ANOVA were applied to compare the scores acquired by students in the two groups of virtual and traditional instruction. The Greenhouse-Geisser correction was applied since the sphericity test could not be performed. To eliminate the confounding effect of GPA, ANCOVA was used to analyze the normally distributed data. All statistical analyses were carried out using SPSS version 18 (SPSS Inc., IL, USA) at 0.05 level of significance.

## Results

A total of 39 fourth year dental students with a mean GPA score of 15.4 ± 1.13 participated in this study. The mean GPA score was 15.64 ± 1.19 in the virtual learning and 15.14 ± 1.03 in the traditional learning group. The two groups were not significantly different in terms of their GPA score (*P* = 0.174). There were 7 males (35%) and 13 females (65%) in the virtual learning and 5 males (26.3%) and 14 females (73.7%) in the traditional learning group. The mean age of participants was 24.2 years in the virtual learning and 23.7 years in the traditional learning group. The difference between the two groups was not significant in terms of age or sex (*P* > 0.05).

The Shapiro-Wilk test showed normal distribution of data (*P* > 0.05). Table 1 presents the mean scores acquired by students in the two groups in the first and second theoretical tests. The mean score acquired by students in virtual education group in the theoretical exam was higher than that in the traditional learning group. Also, in both methods of education, the mean score acquired by students in the theoretical exam at 2 months after the instruction was lower than the score acquired in the immediate test. No significant difference was noted between the two groups regarding the scores acquired in the immediate theoretical exam or at 2 months (*P* = 0.224).

**Table 1:** Mean scores acquired by students in the two groups in the first and second theoretical tests

Group	Immediate theoretical test		Theoretical test at 2 months		Difference	
	Mean	SD	Mean	SD	Mean	SD
Virtual learning	16.60	0.91	15.88	0.78	−0.73	0.66
Traditional learning	14.89	0.99	14.45	0.83	−0.45	0.74
*P* value	< 0.001		< 0.001		0.224	

*SD* Standard deviation

Repeated measures ANOVA showed that type of education (virtual versus traditional) had a significant effect on the theoretical test score of students (F = 36.568, *P* < 0.001). Time had a significant effect on the theoretical test score of students as well (F = 27.248, P < 0.001). However, the interaction effect of time and type of education was not significant on the theoretical test score of students (F = 1.528, P = 0.224).

Table 2 shows the mean scores acquired by students in the two groups in the first and second OSCEs. The mean OSCE score of students in virtual education group was higher than that of students in traditional education group. Also, in both methods of education, the mean score acquired by students in the OSCE at 2 months after the instruction was lower than the score acquired in the immediate exam. No significant difference was noted between the two groups regarding the scores acquired in the immediate OSCE or at 2 months (*P* = 0.425).

**Table 2:** Mean scores acquired by students in the two groups in the first and second clinical exams (OSCE)

Group	Immediate clinical exam		Clinical exam at 2 months		Difference	
	Mean	SD	Mean	SD	Mean	SD
Virtual learning	15.13	0.78	14.75	0.87	−0.38	0.65
Traditional learning	14.71	0.92	14.18	0.95	−0.53	0.51
P value	0.136		0.059		0.425	

*SD* Standard deviation

Repeated measures ANOVA revealed that type of education (virtual versus traditional) had no significant effect on the OSCE score of students (F = 3.422, *P* = 0.072). Time had a significant effect on the OSCE score of students (F = 23.107, *P* < 0.001). The interaction effect of time and type of education on the OSCE score of students was not significant (F = 0.651, P = 0.425).

## Discussion

The results showed that the mean scores of theoretical test and OSCE in the virtual education group were higher than those in the traditional education group. In both methods of instruction, the mean scores of theoretical test and OSCE at 2 months were lower than the scores acquired immediately after instruction but this difference was not statistically significant. Type of education had a significant effect on the theoretical test score of students but had no significant effect on the clinical test score.

Virtual learning is becoming increasingly popular in medical education [24]. A study conducted in Switzerland evaluated the efficacy of blended learning for acquisition of basic surgical techniques and reported that blended learning successfully enhanced cognition, performance and training efficiency [25]. Mitchell et al. [26] reported that nursing students who had frequent access to electronic educational content gained higher scores in exams. Nourian et al. [27] compared the efficacy of electronic learning and traditional classroom instruction for theoretical course of dental public health and found that both methods were equality effective in terms of knowledge acquisition. Nikzad et al. [28] and Aragon and Zibrowski [29] reported that supplemental methods such as educational films and study guides were effective for instruction of a fixed partial denture course. Kavadella et al. [18] reported that blended learning was significantly effective for instruction of oral and maxillofacial radiology topics.

A review study on articles regarding continuing medical education via virtual and traditional learning published from 1996 to 2004 showed that virtual learning was as effective as traditional learning in most studies but six studies reported superior efficacy of virtual learning [28]. Hugenholtz et al. [30] demonstrated that both virtual and traditional learning methods for continuing medication education effectively enhanced the knowledge of physicians with no significant difference. Moazami et al. [21] compared the effects of virtual versus traditional education in endodontics on learning of dental students. They compared the mean knowledge score of both groups and revealed that virtual learning was more effective than traditional learning. Similarly, our study showed that students in virtual education group gained significantly higher scores in the theoretical exam compared with the traditional education group. The virtual education group also gained a higher score in OSCE compared with the traditional group but this difference did not reach statistical significance. This finding is also in agreement with the results of Belcher et al. [31] who stated that easy access to educational content in virtual education results in higher satisfaction of learners and consequently improved learning. It should be noted that they evaluated the nursing staff education in their study. Leasure et al. [32] in their study on a nursing research course for nursing students found that virtual instruction is more effective than classroom education by 19%. In a study by Ludlow et al. [33] dental students mentioned that easy access to educational content and high-quality of radiographs were among the factors that helped in achieving their educational goals. Meckfessel et al. [9] decreased the rate of failure of dental students in the final exams of dental radiology from 40 to 2% by employing virtual education in comparison with traditional education (control group). Sendra-Portero et al. [15] concluded that virtual education can be used as an alternative to traditional education for instruction of radiology contents to medical students, without negatively affecting their learning.

Some studies are also available reporting the superiority of traditional learning to virtual instruction [24, 34]. This result is different from our findings and may be attributed to the unavailability of equipment, absence of the required infrastructure, different level of interest of students in the taught topic, or absence of supportive measures such as group discussions, which may decrease the motivation of students. In the present study, we tried to compensate for the lack of group discussion in virtual learning by creating a forum in our website allowing the students to interact and communicate with each other and also with the mentor and discuss the topics of each session. This may explain the difference between our findings and those of some previous studies such as the ones by Thurmond [24] and Khatoni et al. [34].

Most previous studies on virtual education have evaluated and compared the efficacy of virtual and traditional education of theoretical topics. One major strength of the current study was that we evaluated both the theoretical knowledge and reporting skills gained by students with regard to radiographic interpretation of bony lesions of the jaw via the two educational methods. A group of faculty members of the Oral and Maxillofacial Radiology Department of Shiraz University confirmed the validity of the theoretical test and OSCE. Also, reliability of the tests was assessed by KR20 test. This was another strength of the current study since most of the previous studies on this topic did not assess the validity and reliability of their tests.

Small sample size (evaluation of fifth year dental students of one dental school) was a limitation of this study. Inequality in computer knowledge and skills of students may be considered as another limitation of this study. However, we held an informatory session on how to use LMS to minimize this difference.

Future multi-center studies with larger sample size are required to compare the efficacy of virtual and traditional instruction of theoretical and clinical courses. Also, the efficacy of blended learning for instruction of clinical courses needs to be evaluated in future studies.

## Conclusions

The virtual method was more effective than the traditional method for instruction of radiographic interpretation of bony lesions of the jaw. However, this superiority was greater for the theoretical aspect of the topic. Considering the superiority of virtual method for teaching of theoretical topics and its equal efficacy with the traditional method for instruction of clinical reporting skills, virtual education can serve as an effective alternative to traditional classroom teaching for teaching of radiographic interpretation of bony lesions of the jaw to dental students.

## Additional files

Additional file 1: Theoretical test. (DOCX 31 kb)

Additional file 2: OSCE. (PPSX 3467 kb)

## Data Availability

All materials described in this manuscript including all relevant raw data, will be freely available to any scientist wishing to use them for non-commercial purposes, without breaching participant confidentiality. The data of this research is available from Ehsan Bahrampour (corresponding author).
