# Supplementary material for: Effect of virtual versus traditional education on theoretical knowledge and reporting skills of dental students in radiographic interpretation of bony lesions of the jaw
Source: BMC Med Educ. 2019 Jun 25;19:233. doi: 10.1186/s12909-019-1649-0 (PMC6593487; doi:10.1186/s12909-019-1649-0)
Supplement: Supplementary file 1 — Theoretical test. (DOCX 31 kb) [file 12909_2019_1649_MOESM1_ESM.docx]

**Theoretical exam: Name: Family name:**

1. Which of the following shows the correct order of analysis of bone lesions from left to right?
2. Assess the periphery and shape, (B) analyze the internal structure, (C) formulate interpretation, (D) localize abnormality, (E) analyze the effects of lesion on the surrounding structures
3. A>B>C>D>E
4. D>C>B>A>E
5. D>A>B>E>C
6. C>D>A>B>E
7. Which statement is incorrect regarding the epicenter of lesions with respect to their location?
8. If the epicenter of lesion is in the coronal region of the tooth, it has been probably originated from the odontogenic epithelium.
9. If the lesion is under the inferior alveolar nerve, it has been probably originated from the odontogenic epithelium.
10. If the lesion is within the inferior alveolar nerve canal, it probably has a neurovascular origin.
11. If the lesion is in the maxillary sinus, it does not have an odontogenic origin.
12. Upward displacement of the inferior alveolar nerve is a major characteristic of which of the following lesions?
13. Neurovascular lesions
14. Malignant neoplasms
15. Fibrous dysplasia
16. Osteomyelitis
17. Definition of which of the following lesions is different from others’?
18. Refractory osteitis
19. Sclerosing osteitis
20. Condensing osteitis
21. Focal sclerosing osteitis
22. Which statement is incorrect regarding acute osteomyelitis?
23. It affects all age groups.
24. Has a strong predilection for males
25. Its origin is infectious.
26. It has equal prevalence in the maxilla and mandible

1. Which statement is true regarding radiographic examination in acute phase of osteomyelitis?
2. Positive technetium bone scan indicates infiltration of inflammatory cells.
3. Positive technetium bone scan positive technetium bone scan indicates increased metabolic activity of bone.
4. Positive gallium citrate bone scan indicates decreased metabolic activity of bone.
5. Positive gallium citrate bone scan indicates increased metabolic activity of bone.

1. The punched-out appearance has a …….and …….. margin and is seen in …….
2. Wide and radiopaque/periapical dysplasia
3. Sharp and distinct/multiple myeloma
4. Thin and radiopaque/cysts
5. Lucent and encapsulated/cementoblastoma
6. Inflammation of soft tissues surrounding the crown of semi-erupted mandibular third molars is called…..
7. Osteomyelitis
8. Condensing osteitis
9. Acute periapical abscess
10. Pericoronitis
11. Which one is the most common cyst of the jaws?
12. Radicular cyst
13. Residual cyst
14. Dentigerous cyst
15. Nasolabial cyst
16. A lucent lesion was accidentally noted on a panoramic radiograph of a patient requiring dental treatment surrounding the crown of a third molar from CEJ to CEJ. The patient had no pain or discomfort. The radiolucency had a well-defined round margin and had displaced the involved tooth apically causing distal root resorption of the adjacent second molar. What is your diagnosis?
17. Ameloblastic fibroma
18. Radicular cyst
19. Dentigerous cyst
20. Buccal bifurcation cyst
21. Which one is true regarding keratocystic odontogenic tumor?
22. It often causes severe swelling of the jaws.
23. It is more common in the anterior regions of the maxilla and mandible.
24. It has a dominantly radiopaque appearance inside.
25. Its extension is often greater than its expansion.
26. Which cyst is not often detected on the conventional radiographs (panoramic, periapical) and is only detectable by CT or MRI?
27. Nasolabial cyst
28. Nasopalatine duct cyst
29. Glandular odontogenic cyst
30. Simple bone cyst
31. Which one of the following odontogenic tumors has an epithelial origin?
32. Ameloblastic fibroma
33. Ameloblastoma
34. Ameloblastic fibro-odontoma
35. Odontoma
36. Granular or wispy septae are seen in ……
37. Odontogenic myxoma
38. Central giant cell granuloma
39. Ossifying fibroma
40. Ameloblastoma
41. Which statement is true regarding odontoma?
42. The site of mandibular first and second molars is the most common site of compound odontoma.
43. The prevalence of complex odontoma is higher than compound odontoma.
44. The internal structure of complex odontoma includes an unidentified mass of calcified tissue.
45. Dilated odontoma is the mildest manifestation of dense in dent.
46. Which of the followings is not a symptom of McCune-Albright syndrome?
47. Café au lait spots
48. Monostotic fibrous dysplasia
49. Endocrine gland hyper-function
50. Polyostotic fibrous dysplasia
51. In involvement of the maxillary sinus by fibrous dysplasia, extension of lesion into the sinus often occurs from the …. wall and the ….. area is the last region of the sinus that is involved.
52. Superior-lateral/lateral
53. Lateral/medial
54. Posterior-inferior/anterior
55. Lateral/posterior-superior
56. Which lesion has a higher possibility to be accompanied by traumatic bone cyst?
57. Periapical cemental dysplasia, Florid osseous dysplasia
58. Cemento-ossifying fibroma , Periapical cemental dysplasia
59. Cemento-ossifying fibroma , Florid osseous dysplasia
60. Central giant cell granuloma , Periapical cemental dysplasia
61. Which one is the most common clinical presentation of central giant cell granuloma?
62. Painless swelling
63. Pain
64. Purple color of the covering mucosa
65. Very fast growth
66. If the internal structure of central giant cell granuloma is granular, what condition would be in its list of differential diagnoses?
67. Cemento-ossifying fibroma
68. Ameloblastoma
69. Odontogenic myxoma
70. Aneurysmal bone cyst
71. A 25-year-old female presented complaining of pain at the mandibular right molar region. A round lesion with a multi-locular pattern and ill-defined wispy septae was noted on her radiograph. The septae had 90° angle relative to the expanded external border. What is your diagnosis?
72. Ameloblastoma
73. Hemangioma
74. Aneurysmal bone cyst
75. Odontogenic myxoma
76. Anterior displacement of posterior teeth is pathognomonic for which of the following lesions?
77. Basal cell nevus syndrome
78. Fibrous dysplasia
79. Langerhans cell disease
80. Cherubism
81. A 65-year old hunched-back male was presented complaining of skeletal pains and denture misfit. The serological tests showed very high level of serum alkaline phosphatase. What is your primary diagnosis?
82. Osteogenic sarcoma
83. Paget disease
84. Multiple myeloma
85. Langerhans cell histiocytosis
86. Which area is not a common site for development of squamous cell carcinoma with soft tissue origin?
87. Lateral borders of the tongue
88. Lingual part of the posterior mandible
89. Hard palate
90. Lip and floor of the mouth
91. Which area is not a common location for chondrosarcoma?
92. Coronoid process
93. Condylar head
94. Mandibular symphysis
95. Posterior maxilla
96. Which statement is true regarding Ewing sarcoma?
97. Root resorption of involved teeth is common.
98. It is radiolucent and has a cortical border.
99. Radiotherapy and chemotherapy play no role in its treatment.
100. Rarely involves the jaws.
101. Which one is the most common skeletal malignancy in adults?
102. Multiple myeloma
103. Burkitt’s lymphoma
104. Ameloblastoma
105. Ewing sarcoma
106. Which statement is not true regarding the Stafne bone cyst?
107. It is a pseudo-cyst
108. It creates a round, well-defined radiolucency.
109. It is commonly seen in submandibular and sublingual salivary glands.
110. It is often located above the inferior alveolar nerve canal.
111. The cluster of small pebbles pattern is seen in…..
112. CEOT
113. AOT
114. COF
115. COC
116. Multiple radiolucencies with ill-defined borders, no cortical margin and balloon-shaped swelling are seen in….
117. Ewing sarcoma
118. Leukemia
119. Burkitt’s lymphoma
120. Osteosarcoma
121. Pericoronitis causes periosteal stimulation for bone formation in which of the following lesions?
122. Inferior cortex, buccal cortex and angle of mandible
123. Buccal cortex, posterior border of ramus and angle of mandible
124. Along the coronoid notch, inferior cortex and posterior border of ramus
125. Along the coronoid notch, angle of mandible and posterior border of ramus
126. Which statement is not true regarding osteoradionecrosis?
127. It is more common in the mandible
128. Its radiographic manifestation is similar to the chronic phase of osteomyelitis.
129. The most common effect of osteoradionecrosis on the surrounding bone is stimulation of sclerosis.
130. A wide range from bone destruction to bone formation is seen and destruction is dominant to formation.
131. Which statement is not true regarding bisphosphonate-related osteonecrosis of the jaw?
132. Patients often show an area of exposed bone clinically after a dental surgical procedure.
133. Involvement of both jaws is the most common type of involvement.
134. There is no pathognomonic radiograph sign in most cases.
135. Increased thickness of lamina dura can be one of its radiographic signs.
136. Which one is the most important differential diagnosis for incisive canal cyst?
137. Large incisive foramen
138. Radicular cyst
139. Nasolabial cyst
140. Dermoid cyst
141. Severe expansion in OKC has a higher likelihood to involve the….
142. Lower ramus and coronoid process
143. Condyloid process and coronoid process
144. Upper ramus and coronoid process
145. Condyloid process and lower ramus
146. The most common characteristic of a buccal bifurcation cyst is elongation of the involved molar root apex towards the …… cortical plate and …. is the best diagnostic radiography for its detection.
147. Buccal/occlusal cross-sectional radiography of the mandible
148. Lingual/CT
149. Buccal/CT
150. Lingual/occlusal cross-sectional radiography of the mandible
151. Which one is the dominant site of occurrence of lateral periodontal cyst?
152. Between the mandibular lateral incisor to second premolar
153. Between the mandibular lateral incisor and canine
154. Between the maxillary lateral incisor to second premolar
155. Between the maxillary lateral incisor and canine
156. Which one has a lower likelihood to be included in the list of differential diagnoses for glandular odontogenic cyst?
157. Ameloblastoma
158. OKC
159. Mucoepidermoid carcinoma
160. Adenoid cystic carcinoma
161. The spokes of a wheel pattern is seen in …..
162. Hypercementosis
163. Simple bone cyst
164. Fibrous dysplasia
165. Healing radicular cyst
166. Which one of the following can help in differentiation of a dentigerous cyst from a hyperplastic follicle?
167. Tooth displacement
168. Margin
169. Bone swelling
170. Internal structure
171. I and II
172. I and III
173. III and IV
174. I and IV
