# Supplementary figures and images for: Effect of virtual versus traditional education on theoretical knowledge and reporting skills of dental students in radiographic interpretation of bony lesions of the jaw
Source: BMC Med Educ. 2019 Jun 25;19:233. doi: 10.1186/s12909-019-1649-0 (PMC6593487; doi:10.1186/s12909-019-1649-0)

## Slide 1
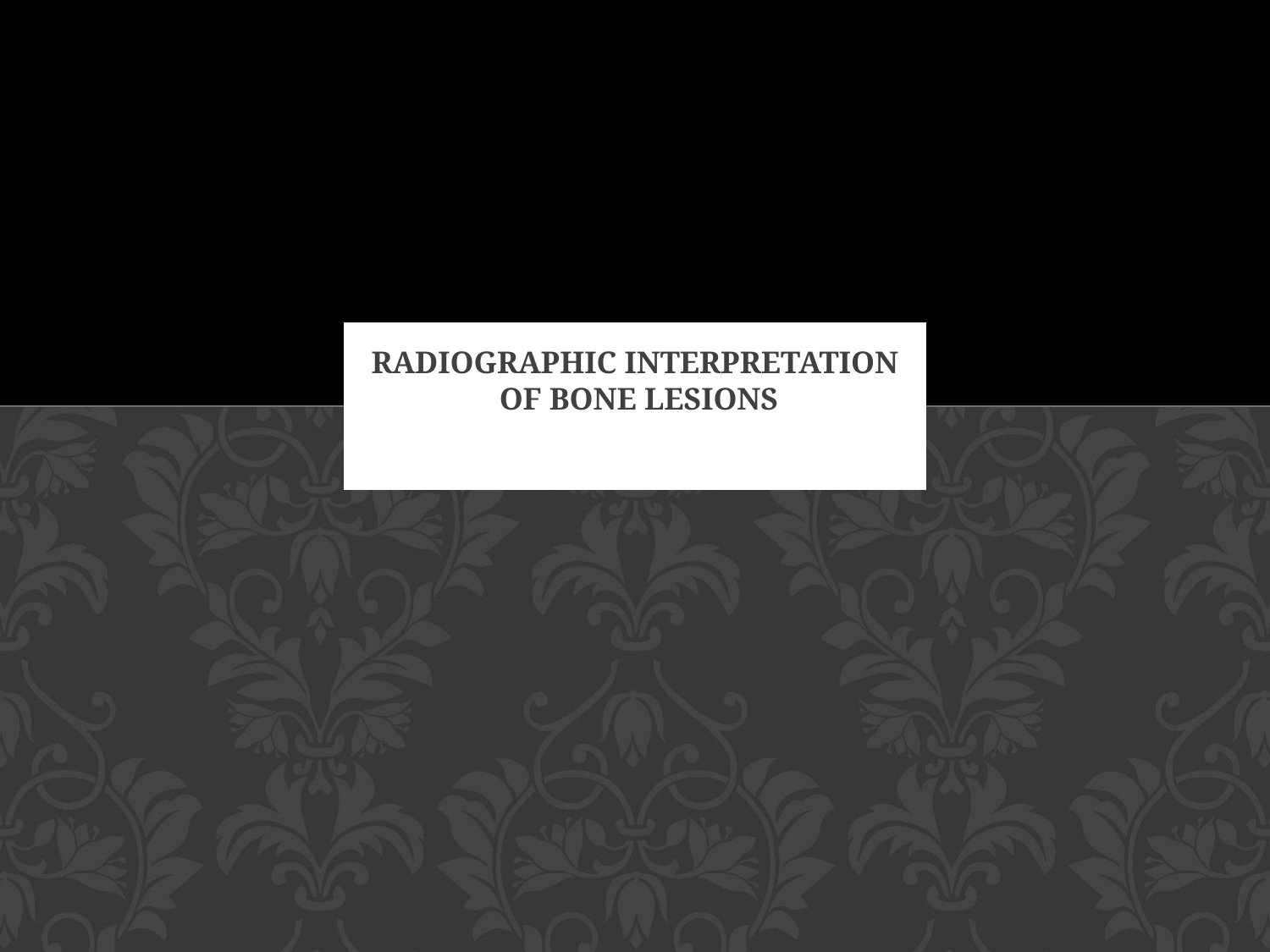

## Slide 2
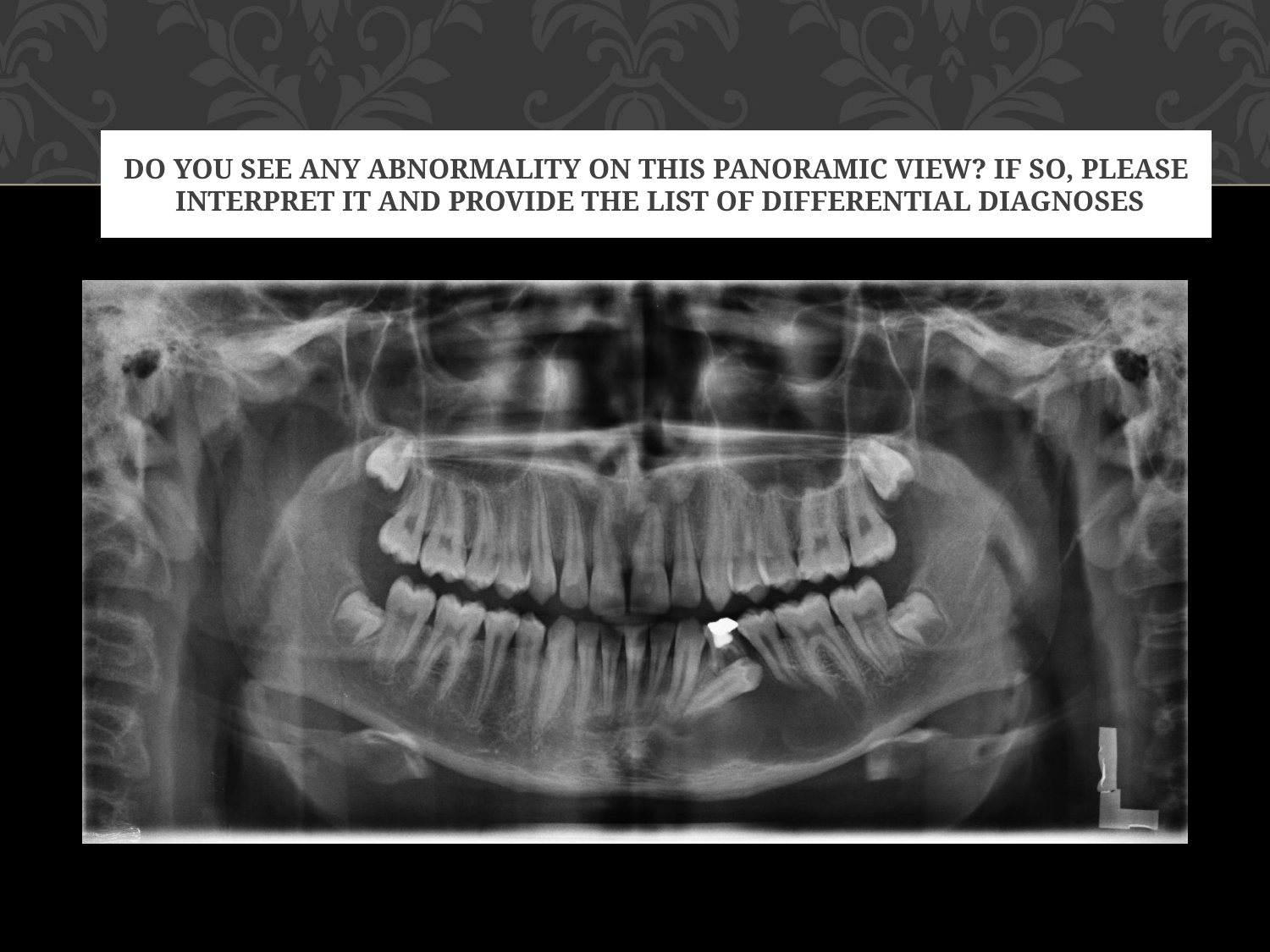

## Slide 3
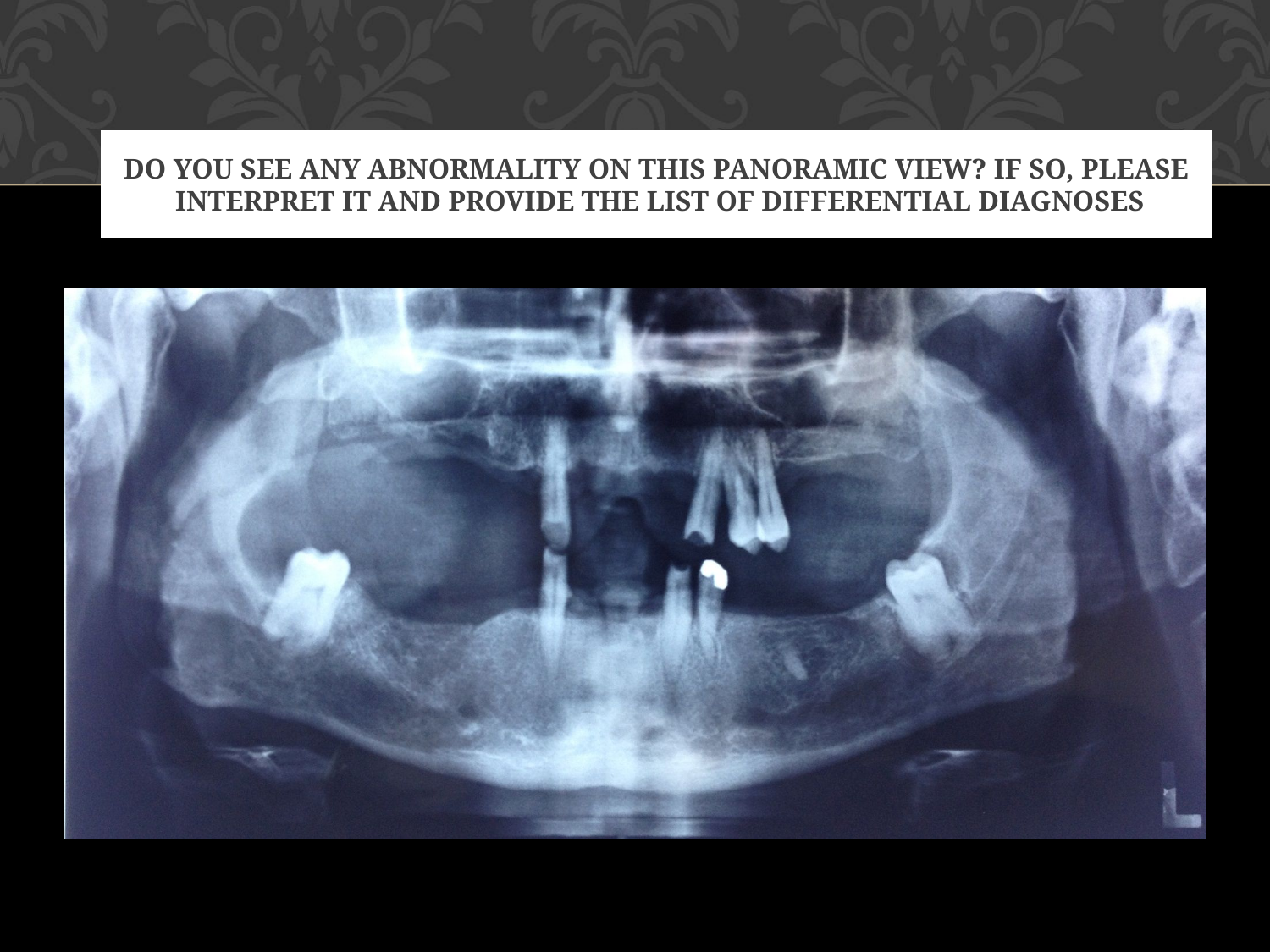

## Slide 4
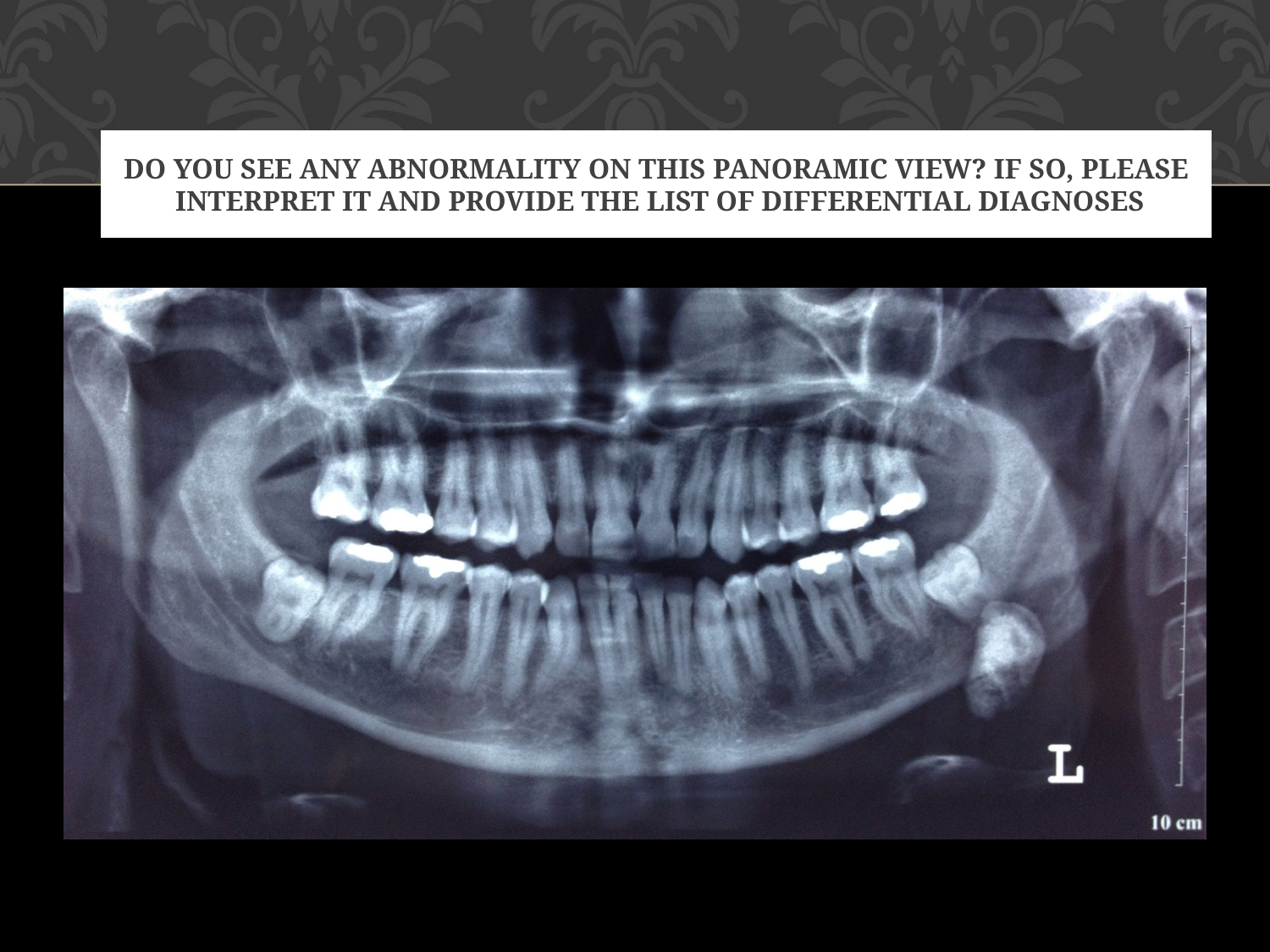

## Slide 5
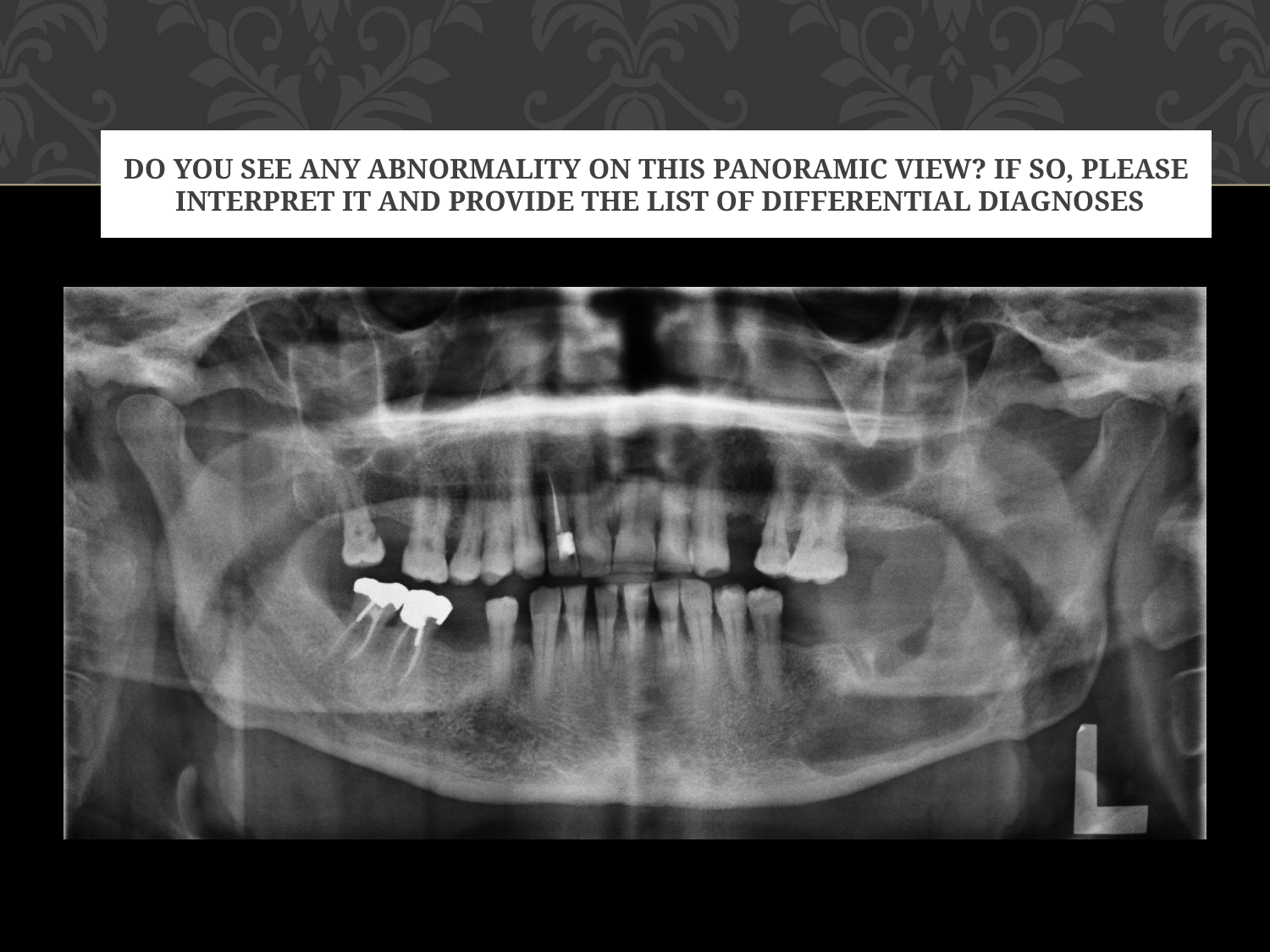

## Slide 6
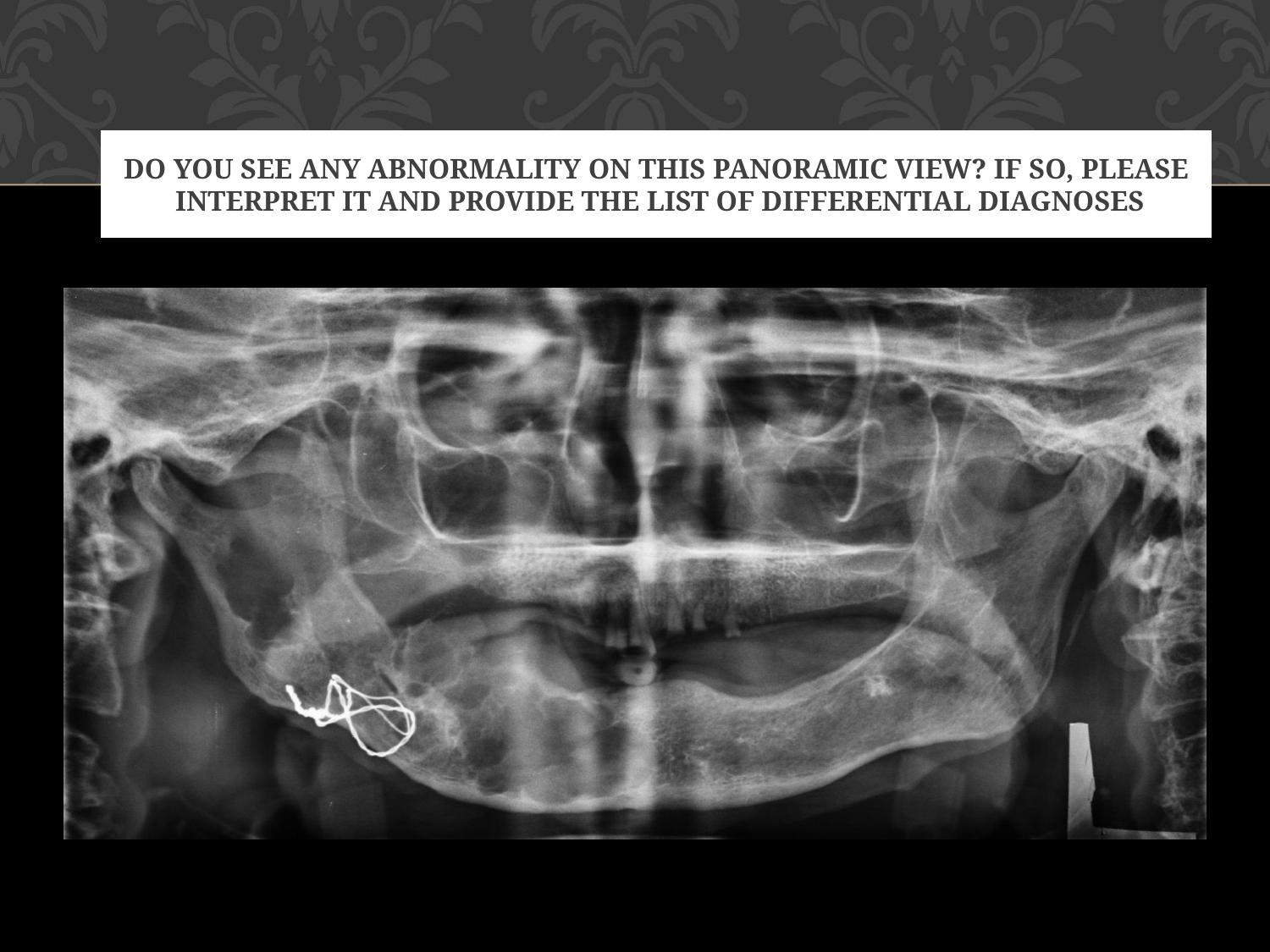

## Slide 7
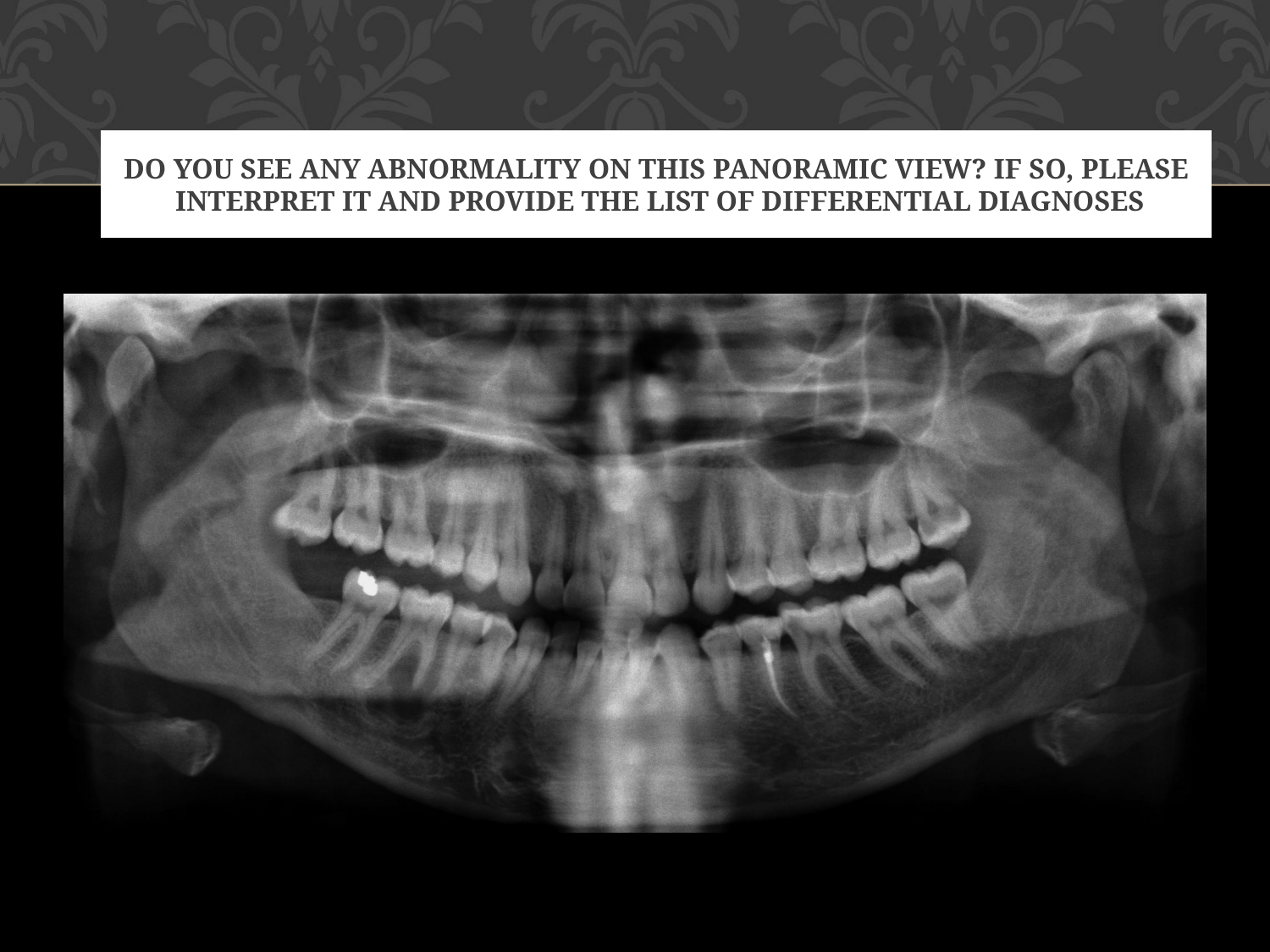

## Slide 8
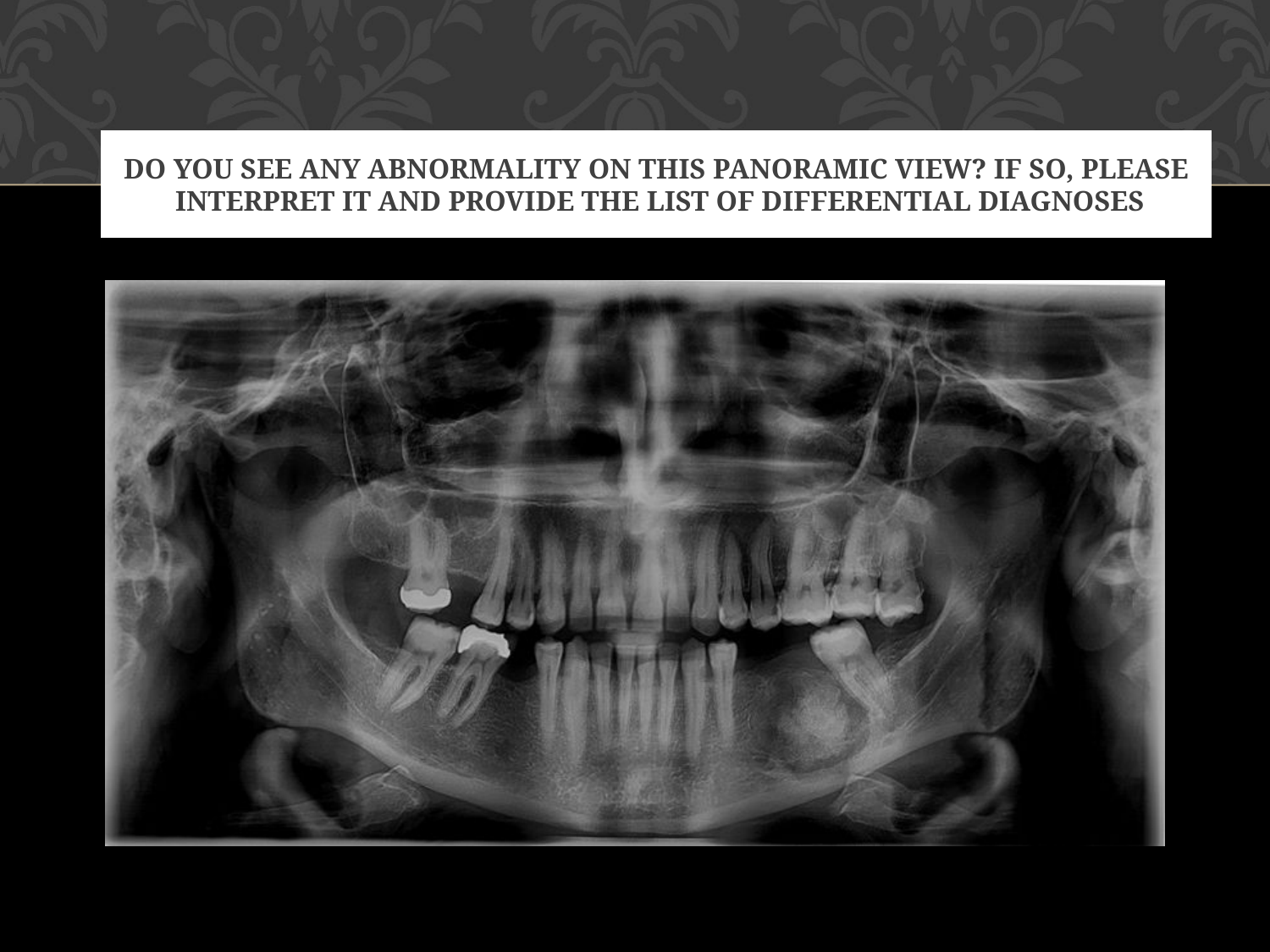

## Slide 9
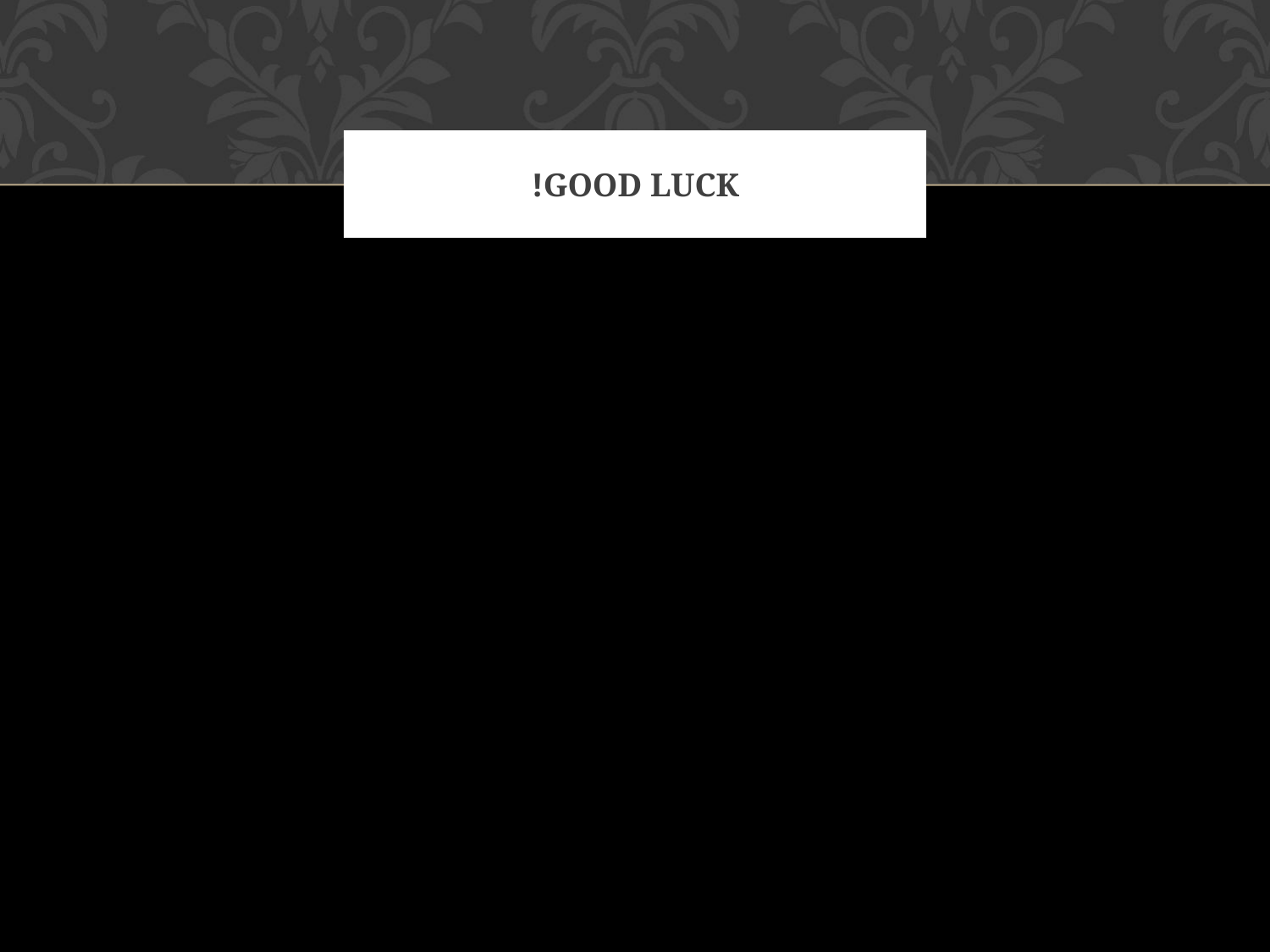

Supplement: Supplementary file 2 — OSCE. (PPSX 3467 kb) [file 12909_2019_1649_MOESM2_ESM.ppsx]
